# Spontaneous Coronary Artery Dissection with Cardiogenic Shock in the United States

**DOI:** 10.31083/j.rcm2503086

**Published:** 2024-03-05

**Authors:** Chayakrit Krittanawong, Dhrubajyoti Bandyopadhyay, Neelkumar Patel, Yusuf Kamran Qadeer, Neil Sagar Maitra, Zhen Wang, Mahboob Alam, Samin Sharma, Hani Jneid

**Affiliations:** ^1^Cardiology Division, NYU Langone Health and NYU School of Medicine, New York, NY 10016, USA; ^2^Department of Cardiology, Massachusetts General Hospital (MGH), Harvard Medical School, Boston, MA 02124, USA; ^3^Department of Cardiology, Maimonides Medical Center, Brooklyn, NY 11219, USA; ^4^Section of Cardiology, Baylor College of Medicine, Houston, TX 77030, USA; ^5^Division of Cardiology, Scripps Clinic, La Jolla, CA 92037, USA; ^6^Robert D. and Patricia E. Kern Center for the Science of Health Care Delivery, Mayo Clinic, Rochester, MN 55902, USA; ^7^Division of Health Care Policy and Research, Department of Health Sciences Research, Mayo Clinic, Rochester, MN 55902, USA; ^8^The Texas Heart Institute, Baylor College of Medicine, Houston, TX 77030, USA; ^9^Cardiac Catheterization Laboratory of the Cardiovascular Institute, Mount Sinai Hospital, New York, NY 10018, USA; ^10^Division of Cardiology, University of Texas Medical Branch, Houston, TX 77030, USA

**Keywords:** SCAD, cardiogenic shock, shock

## Abstract

**Background:**

Spontaneous coronary artery dissection (SCAD) is defined as a non-traumatic separation of the epicardial coronary artery walls that creates a false lumen. SCAD poses a difficult challenge in management, as decisions regarding revascularization and medical management seem to be tailored to the individual patient. We evaluated and compared outcomes based on cardiogenic shock in patients with SCAD utilizing Nationwide Readmissions Database (NRD) between January 1, 2016, to December 30, 2020.

**Methods:**

We utilized the NRD 2016–2019 to carry out this study. We evaluated demographics (e.g., age, gender), conventional risk factors, comorbidities present on the index admission, and in-hospital outcomes using their specific ICD-10-CM codes. The primary outcomes were In-hospital mortality and 30-day readmission, and the secondary outcome was to compare the complications in SCAD patient with cardiogenic shock (CS) compared to those without CS.

**Results:**

We analyzed 2473 individuals with SCAD, 2199 of these individuals did not have cardiogenic shock whereas 274 of these individuals did have cardiogenic shock. When comparing SCAD with cardiogenic shock to SCAD without cardiogenic shock, there was a statistically significant increased odds ratio (OR) for death (propensity matched OR 24.93 (7.49–83.05), use of mechanical circulatory support (propensity matched OR 15.30 (6.87–34.04), ventricular tachycardia (propensity matched OR 4.45 (1.92–10.34), utilization of blood transfusions (propensity matched OR 3.82 (1.86–7.87), acute kidney injury (propensity matched OR 4.02 (1.45–11.13), need for mechanical ventilation (propensity matched OR 8.87 (3.53–22.31), and respiratory failure (propensity matched OR 4.95 (1.83–13.41)))))))). There was no statistically significant difference in 30-day readmission rates between the two groups.

**Conclusions:**

SCAD is a unique condition that can lead to many complications. In our analysis, we showed that SCAD associated with cardiogenic shock compared to SCAD not associated with cardiogenic shock results in greater odds of complications including death, use of mechanical circulatory support, need for blood transfusions, ventricular tachycardia, acute kidney injury, use of mechanical ventilation, and respiratory failure. SCAD with cardiogenic shock represents a significantly critical clinical scenario that requires a multi-disciplinary approach to prevent the many potential complications associated with this disease process.

## 1. Introduction

Spontaneous coronary artery dissection (SCAD) is defined as an epicardial coronary artery dissection that is not associated with atherosclerosis or trauma and not iatrogenic [1]. The proposed mechanism of SCAD is coronary artery obstruction caused by formation of an intramural hematoma (most frequent form of SCAD) or intimal disruption [2]. This disease process is more common in younger women, particularly with pregnancy [3]. To date, fibromuscular dysplasia is now not considered as a SCAD risk factor but an associated pathology with probable common genetic background. SCAD poses a difficult challenge in management, as decisions regarding revascularization and medical management seem to be tailored to the individual patient; in general, a conservative strategy seems to be the mainstay of treatment with revascularization dictated by the clinical status of the patient [4, 5]. Cardiogenic shock (CS) incidence varies between 1–16% in patients with SCAD and the outcomes as well as management of these patients still remains a challenge considering rare occurrence. Therefore, we evaluated and compared outcomes (such as mortality, readmission rates, and complications) based on CS in patients with SCAD utilizing Nationwide Readmissions Database (NRD) between January 1, 2016, to December 30, 2020.

## 2. Methods

We analyzed the NRD from 2016–2019. Drawing data from Healthcare Cost and Utilization Project (HCUP) State Inpatient Databases (SID), the NRD used verified patient linkage numbers to track entrants across hospitals within a state. The NRD contains unweighted data from about 18 million discharges each year in the United States and weighted, it estimates approximately 36 million discharges. Patients with the principal diagnosis or primary discharge diagnosis of SCAD with and without CS were identified, defined as ICD-CM 10 codes of I25.42 and R570. We excluded patients for various factors: missing critical demographic information, such as age; missing data for mortality; index admissions in the month of December because 30-day readmission outcomes would not be feasible; age <18; and concomitant iatrogenic puncture or laceration of the coronary vessels (ICD-10-CM code of I97.51). We followed the methodological standards recommended by the Agency for Healthcare Research and Quality.

We evaluated demographics (e.g., age, gender), conventional risk factors, comorbidities present on the index admission, and in-hospital outcomes using their specific ICD-10-CM codes as seen in Table 1. Aggregated Charlson comorbidity score was also calculated [6]. Complications included death, cardiac arrest, mechanical circulatory support (Intra-aortic balloon pump (IABP), impella, extracorporeal membrane oxygenation (ECMO)), cardiovascular implantable electronic device (CIED) use, sepsis, pressor support, stroke, acute kidney injury (AKI), dialysis, acute heart failure (HF), bleeding, and need for blood transfusions. In accordance with the HCUP Data Use Agreement, we excluded any number or variables containing small numbers of observations (≤10) that could potentially pose the risk for identification of persons or data privacy violation.

**Table 1.** **Demographic information**.

		Without cardiogenic shock (%)	With cardiogenic shock (%)
Total population (N)		2199	274
Total population discharged alive		2177	197
Age in years ± SD ^#^		52.83 ± 10.43	59.89 ± 9.74
Mean LOS (Days) ^#^		3.57 ± 2.78	9.38 ± 6.33
Female *		76.55	62.53
Insurance ^#^			
	Medicare (%)	22.44	50.6
	Medicaid (%)	12.59	9.28
	Private (%)	59.92	36.05
	Self-pay (%)	5.05	4.07
Teaching hospitals (%) *		81.62	88.65
Hospital Bed size (%)			
	Small	11.28	9.73
	Medium	25.06	18.54
	Large	63.66	71.73
Hospital volume quintile			
	1	10.27	6.04
	2	11.43	11.54
	3	14.12	13.54
	4	20.78	22.35
	5	43.39	46.54
Patient’s residence			
	Large metropolitan areas with at least 1 million residents	57.33	57.34
	Small metropolitan areas with less than 1 million residents	40.19	40.39
	Micropolitan areas and nonurban residual	2.48	2.26
Died			
Charlsoncat score (%) ^#^			
	0	26.17	8.36
	1	40.1	20.75
	2	19.96	25.43
	3	13.77	45.46
Chronic comorbidities (%)			
	Prior Stroke	3.93	4.41
	Prior MI	13.61	8.11
	Prior PCI *	10.85	20.28
	Prior CABG	3.65	3.76
	Anemia	3.02	2.82
	Pulmonary HTN *	2.03	6.72
	Hypertension *	58.15	71.2
	Dyslipidemia	47.22	52.32
	Metabolic syndrome *	0.18	1.22
	Malignancy *	6.24	1.59
	PVD ^#^	7.02	17.53
	CHF ^#^	17.8	56.25
	Chronic lung disease	15.56	20.79
	DM *	13.61	22.23
	Obesity	24.33	24.96
	OSA	7.6	6.99
	CADAE ^#^	48.25	75.21
	CKD *	4.26	11.72
	Smoking	20.89	19.12
	Alcohol use	1.36	2.54
	Coagulation disease ^#^	4.33	43.18
	Drug use	4.1	1.1
	Hypothyroidism	12.74	13.5
	Electrolytes disturbances ^#^	15.3	64.91

*: *p*-value < 0.05; #: *p*-value < 0.001. LOS, length of hospital stay; MI, myocardial infarction; PCI, percutaneous coronary intervention; CABG, coronary artery bypass grafting; HTN, hypertension; PVD, peripheral vascular disease; CHF, congestive heart failure; DM, diabetes mellitus; OSA, obstructive sleep apnea; CADAE, coronary artery disease and equivalents; CKD, chronic kidney disease.

The primary outcomes were in-hospital mortality and 30-day readmission, and the secondary outcome was to compare the complications in SCAD patient with CS compared to those without CS. The weighting of patient-level observations was implemented to obtain national estimates. Multivariate regression analysis models were built by including all confounders significantly associated with the outcome on univariable analysis with a cutoff *p*-value of 0.2. Variables deemed important determinants of the outcomes based on literature were forced into the models. Binary outcomes such as index hospitalization mortality and complications were compared by logistic regression. For readmission comparison, we used the time-to-event Cox regression analysis. Propensity matching was done by the Greedy method. Proportions were compared using the Mantel-Haenszel Chi-square test or Fisher exact test, and continuous variables were compared using the Student *t*-test. All *p* values were two-sided, with 0.05 as the threshold for statistical significance. Percentages and means were computed for categorical and continuous variables, respectively. Odds/hazard ratios and their 95% confidence intervals are used to report the regression analysis results. All statistical analyses were performed using STATA (Version 16, Stata Statistical Software, College Station, TX, USA).

## 3. Results

We analyzed 2473 individuals with SCAD, 2199 of these individuals did not have cardiogenic shock whereas 274 of these individuals did have cardiogenic shock. As seen in Table 1, the average age of the SCAD without cardiogenic shock group was slightly lower at 53 years compared to the average age of the SCAD with cardiogenic shock group being 60 years. The prevalence of men was higher in the cardiogenic shock group as compared to the non-cardiogenic shock group. The cardiogenic shock group was more likely to have Medicare for their insurance compared to private insurance for the non-cardiogenic shock group. Chronic comorbidities that were more likely to be found in the cardiogenic shock group included pulmonary hypertension, hypertension, peripheral vascular disease, congestive heart failure, diabetes mellitus, coagulation abnormalities, electrolyte imbalances, and coronary artery disease and equivalents (CADAE).

As seen in Table 2, when comparing SCAD with cardiogenic shock to SCAD without cardiogenic shock, there was a statistically significant increased OR for death (propensity matched OR 24.93 (7.49–83.05), use of mechanical circulatory support (propensity matched OR 15.30 (6.87–34.04), ventricular tachycardia (propensity matched OR 4.45 (1.92–10.34), utilization of blood transfusions (propensity matched OR 3.82 (1.86–7.87), acute kidney injury (propensity matched OR 4.02 (1.45–11.13), need for mechanical ventilation (propensity matched OR 8.87 (3.53–22.31), and respiratory failure (propensity matched OR 4.95 (1.83–13.41)))))))). There was no statistically significant difference in 30-day readmission rates between the two groups (Table 3).

**Table 2.** **Index hospital mortality and complication comparison**.

Complications		Without shock (%)	With shock (%)	Multivariate regression analysis	
				Non-propensity matched	Propensity matched
				OR (95% CI) *p*-value	OR (95% CI) *p*-value
Total number		2199	274		
Died		1.02	28.16	33.16 (8.75–125.67) ^#^	24.93 (7.49–83.05) ^#^
Arrest		4.66	17.89	1.24 (0.57–2.68)	1.30 (0.49–3.45)
MCS		5.05	65.88	23.77 (11.65–48.53) ^#^	15.30 (6.87–34.04) ^#^
	IABP	4.66	40.8	10.39 (5.32–20.27) ^#^	6.77 (3.15–14.56) ^#^
	Impella	0.34	21.87	56.20 (8.87–356.09) ^#^	-
	ECMO	0.15	12.64	42.80 (1.87–980) *	-
CIED use		1.2	3.06	0.40 (0.06–2.58)	-
Sepsis		1.34	5.99	0.49 (0.10–2.48)	0.30 (0.07–1.33)
Pressor support		1.26	17.23	5.26 (1.91–14.50) *	4.68 (1.05–20.88) *
VT		9.2	44.59	3.89 (2.28–6.62) ^#^	4.45 (1.92–10.34) *
Acute stroke		0.75	5.98	1.24 (0.26–5.94)	12.42 (1.79–86.11) *
Acute HF		4.68	31.43	2.30 (1.12–4.72) *	2.18 (0.90–5.31) *
Major bleed		0.19	3.96	5.86 (0.81–42.33) *	1.90 (0.12–29.41)
Blood transfusion		1.95	16.54	5.89 (1.71–20.32) *	3.82 (1.86–7.87) ^#^
AKI		4.6	41.86	3.78 (1.82–7.85) ^#^	4.02 (1.45–11.13) *
Dialysis		0.55	9.16	2.08 (0.64–6.79)	-
AKI requiring dialysis		0.18	9.16	6.14 (0.95–39.61)	-
Mechanical ventilation		3.51	55.76	11.12 (5.68–21.77) ^#^	8.87 (3.53–22.31) ^#^
	Less than 24 hours	1.37	19.57	8.74 (3.57–21.40) ^#^	29.61 (5.79–151.53) ^#^
	24–96 hours	1.74	17.7	3.89 (1.32–11.43) *	1.58 (0.59–4.22)
	>96 hours	0.45	20.17	27.17 (4.95–149.28) ^#^	12.21 (0.73–203.87) *
Respiratory failure		5.98	63.82	6.77 (3.61–12.69) ^#^	4.95 (1.83–13.41) *

*: *p*-value < 0.05; #: *p*-value < 0.001. OR, odds ratio; CI, confidence interval; MCS, mechanical circulatory support; IABP, intra-aortic balloon pump; ECMO, extracorporeal membrane oxygenation; CIED, cardiovascular implantable electronic device; VT, ventricular tachycardia; HF, heart failure; AKI, acute kidney injury.

**Table 3.** **30 days readmission comparison**.

30-days readmission	Without shock	With shock	Multivariate regression analysis	
			Non-propensity matched	Propensity matched
			OR (95% CI) *p*-value	OR (95% CI) *p*-value
Total number	195	44	2.00 (0.91–4.40) *	0.74 (0.01–43.8)

*: *p*-value < 0.05. OR, odds ratio; CI, confidence interval.

## 4. Discussion

Data surrounding SCAD have broadened in recent years, however data for SCAD-CS are less robust. The present study is among the largest to evaluate real-world clinical experience of SCAD with comparative evaluation of those with SCAD-CS in both men and women of all ages. In this NRD study, 11% of identified SCAD hospitalizations had cardiogenic shock, which is considerably higher than previous estimates of 2–6% with SCAD-shock [7, 8]. Additionally, SCAD-CS patients were older, less likely to be female, and had higher prevalence of comorbid hypertension (HTN), diabetes mellitus (DM), congestive heart failure (CHF), or existing atherosclerotic cardiovascular disease, a departure from previous findings [8, 9]. Mortality for SCAD-CS was high in our study, as has been described previously with in-hospital mortality rates between 8–30% [8, 9]. Nonetheless, SCAD-CS mortality is likely lower than atherosclerotic MI-related CS [10]. Pharmacologic and mechanical circulatory support were necessary for 17% and 40% of SCAD-CS cases, respectively, and mechanical circulatory support (MCS) strategies included IABP, Impella, and ECMO support.

The reason behind this study’s differing epidemiologic findings is unclear but likely multifactorial. Prior SCAD studies typically compared SCAD populations with non-SCAD (i.e., atherosclerotic) MI populations who represent inherently higher-risk population, or specifically assessed SCAD in women with MI. Our sample included all recorded US hospitalizations for SCAD in the NRD. Further, in our sample those who developed CS were more likely to have high-risk characteristics, which may explain a certain predilection for higher-acuity presentation. Due to limitations of the NRD data and our study, we could not control for potential confounders such as dissection anatomy, thrombolysis in myocardial infarction (TIMI) flow grade, pre-admission medication use (i.e., antiplatelet or anticoagulation therapy), or even misclassification bias (i.e., atherosclerosis-related dissection) that may explain the differences between the SCAD-CS and non-CS groups. Lastly, the variation and potential biases associated with NRD analysis increase the risk of type I error and may account for the differing findings.

Clinical practice recommendations for management of SCAD and in particular SCAD-CS are based on understanding and experience with atherosclerotic coronary disease, which represents an inherently different pathophysiologic phenomenon. Studies have demonstrated spontaneous “healing” of the native coronary vessels after presentation [11, 12], as well as increased technical complexity with PCI for SCAD, which informs the current preference for conservative management. Nonetheless, our experience and understanding are broadening. In a recent observational study of a SCAD population, those treated with PCI at index hospitalization had similar 3-year outcomes compared to those managed medically [13]. Those with high-risk presentation or SCAD-CS are more likely to be treated with revascularization [8]. Additionally, MCS use has been described previously, though largely in case report or case series. Critically ill patients undergo management as deemed medically necessary, and in the largest study of SCAD-CS, 14 patients managed with IABP did not experience IABP-related complications [2]. However, in light of the angiographic course of SCAD, the theoretical risk of iatrogenic vascular injury and/or false lumen propagation that may be associated with MCS strategies require further evaluation. Further data are not available to assess MCS use in SCAD-CS nor to compare supportive strategies utilizing inotropic support alone. Finally, optimal management strategies in cases of recurrent SCAD are even more elusive.

It can be argued that the greater complications seen in SCAD with cardiogenic shock is secondary to both the acute clinical instability of the patient, in addition to these patient’s having significant comorbidities in greater frequency, such as hypertension, congestive heart failure, diabetes mellitus, and peripheral vascular disease, when compared to SCAD without cardiogenic shock. More research needs to be conducted to elucidate other reasons for the greater rate of complications as other factors, such as hospital capabilities and physician experience can also factor in.

## 5. Limitations

The present study contains several limitations, including those noted above. First, the inherent pitfalls of retrospective, non-randomized database analysis cannot be avoided. We performed multivariate regression analysis which was further balanced by propensity matching to adjust for confounding variables, but other confounding factors may be present and unaccounted for. Second, the NRD data are based on ICD-10 codes which are subject to errors or variation in coding practice that cannot be adjusted for. Third, we included patient with SCAD as primary diagnosis for admission, which represents ultimate outcome but does not adequately provide information surrounding initial presentation severity nor clinical progression throughout the stay as data are finalized at the time of discharge. Fourth, the NRD dataset is limited as it includes only hospitalization information rather than individual-level data, without longitudinal follow-up data to allow for longer-term outcome assessment. Fifth, Procedure-level data, such as angiographic and intravascular imaging findings, and detailed management-related information are also not provided. Despite the limitations, our data provide novel findings of the clinical characteristics of SCAD-CS. However, these findings require further assessment through robust prospective study, if feasible. Additionally, management strategies should be evaluated to improve in-hospital and long-term outcomes in this population.

## 6. Conclusions

SCAD is a unique condition that can lead to many complications. In our analysis, we showed that SCAD associated with cardiogenic shock compared to SCAD not associated with cardiogenic shock results in greater odds of complications including death, use of mechanical circulatory support, need for blood transfusions, ventricular tachycardia, acute kidney injury, use of mechanical ventilation, and respiratory failure. It can be argued that the greater complications seen in SCAD with cardiogenic shock is secondary to both the acute clinical instability of the patient, in addition to these patient’s having significant comorbidities in greater frequency, such as hypertension, congestive heart failure, diabetes mellitus, and peripheral vascular disease, when compared to SCAD without cardiogenic shock. Regardless, SCAD with cardiogenic shock represents a significantly critical clinical scenario that requires a multi-disciplinary approach to prevent the many potential complications associated with this disease process.

## Data Availability

The datasets used and/or analyzed during the current study are available from the corresponding author on reasonable request.
